# Glutamine Synthetase and Glutamate Synthase Family Perform Diverse Physiological Functions in Exogenous Hormones and Abiotic Stress Responses in *Pyrus betulifolia* Bunge (*P.be*)

**DOI:** 10.3390/plants13192759

**Published:** 2024-10-01

**Authors:** Weilong Zhang, Shuai Yuan, Na Liu, Haixia Zhang, Yuxing Zhang

**Affiliations:** 1 College of Horticulture, Hebei Agricultural University, Baoding 071001, China; 18322711826@163.com (W.Z.); 13409298238@163.com (S.Y.); lnln82@163.com (N.L.); 2 Pear Technology and Innovation Center of Hebei Province, Baoding 071001, China

**Keywords:** abiotic stress, GOGAT, GS, hormonal response, physiological functions, pear

## Abstract

The unscientific application of nitrogen (N) fertilizer not only increases the economic input of pear growers but also leads to environmental pollution. Improving plant N use efficiency (NUE) is the most effective economical method to solve the above problems. The absorption and utilization of N by plants is a complicated process. Glutamine synthetase (GS) and glutamate synthase (GOGAT) are crucial for synthesizing glutamate from ammonium in plants. However, their gene family in pears has not been documented. This study identified 29 genes belonging to the GS and GOGAT family in the genomes of *Pyrus betulaefolia* (*P.be*, 10 genes), *Pyrus pyrifolia* (*P.py*, 9 genes), and *Pyrus bretschneideri* (*P.br*, 10 genes). These genes were classified into two GS subgroups (GS1 and GS2) and two GOGAT subgroups (Fd–GOGAT and NADH–GOGAT). The similar exon–intron structures and conserved motifs within each cluster suggest the evolutionary conservation of these genes. Meanwhile, segmental duplication has driven the expansion and evolution of the GS and GOGAT gene families in pear. The tissue–specific expression dynamics of *PbeGS* and *PbeGOGAT* genes suggest significant roles in pear growth and development. Cis–acting elements of the GS and GOGAT gene promoters are crucial for plant development, hormonal responses, and stress reactions. Furthermore, qRT–PCR analysis indicated that *PbeGSs* and *PbeGOGATs* showed differential expression under exogenous hormones (GA_3_, IAA, SA, ABA) and abiotic stress (NO_3_^−^ and salt stress). In which, the expression of *PbeGS2.2* was up–regulated under hormone treatment and down–regulated under salt stress. Furthermore, physiological experiments demonstrated that GA_3_ and IAA promoted GS, Fd–GOGAT, and NADH–GOGAT enzyme activities, as well as the N content. Correlation analysis revealed a significant positive relationship between *PbeGS1.1*, *PbeGS2.2*, *PbeNADH*–*GOGATs*, and the N content. Therefore, *PbeGS1.1*, *PbeGS2.2*, and *PbeNADH*–*GOGATs* could be key candidate genes for improving NUE under plant hormone and abiotic stress response. To the best of our knowledge, our study provides valuable biological information about the GS and GOGAT family in the pear for the first time and establishes a foundation for molecular breeding aimed at developing high NUE pear rootstocks.

## 1. Introduction

Nitrogen (N) is essential for plant growth as it forms amino acids, the building blocks of proteins and enzymes. Plants absorb N from the soil, primarily in the forms of nitrate (NO_3_^−^) and ammonium (NH_4_^+^) [1,2]. Most plants absorb NO_3_^−^, which then undergoes a two–step reduction reaction to NH_4_^+^, catalyzed by nitrate reductase (NR) and nitrite reductase (NiR) [3,4]. In the roots, NH_4_^+^ is combined with glutamate and assimilated into glutamine via glutamine synthetase (GS) [5]. Subsequently, glutamate synthase (GOGAT) transfers an amino group from glutamine to 2–oxoglutarate (2–OG) to synthesize glutamate. Thus, the GS/GOGAT cycle is crucial for nitrogen assimilation in plants [6].

As the key enzyme for primary N assimilation, GS exists in two forms: cytosolic (GS1) and chloroplastic (GS2), differentiated by size and subcellular localization [7]. GS1 genes belong to a small multigene family, while GS2 was encoded by 1 to 2 genes [8]. In *Pisum sativum*, three active *GS1*, *GS3A*, and *GS3B* genes have been characterized [9]. In pumpkin and cucumber, seven and four *GS* gene family members were identified, respectively [10]. GS–encoded isoenzymes play crucial roles in plant growth [11]. For instance, overexpression of *GS* in poplar increases enzyme activity and N content [12], with the *OsGS1.1* isoenzyme in rice significantly affecting growth and grain filling [13]. Conversely, *GS1* knockout inhabited the growth and grain yield in maize [14].

Furthermore, *GS* expression exhibited a complex pattern affected by factors such as IAA, GA, cold, and N stress [11,15,16,17,18]. Meanwhile, GOGAT was divided into NADH–GOGAT and Fd–GOGAT based on electron donors [19]. GOGAT family members have been identified in *Arabidopsis* [20], rice [21], poplar [22], tomato, and grape [23]. GOGAT expression is affected by Na_2_CO_3_ and light [22,24]. Interestingly, mutations/deletions in *GOGAT* genes alter plant phenotypes. For example, in rice, *GOGAT* suppression reduced dry weight, chlorophyll content, and enzyme activity [25]. *Fd*–*GOGAT* mutants in barley and Arabidopsis (gls) exhibited severe chlorosis and growth defects [26,27]. In transgenic alfalfa and Arabidopsis glt1–t mutants, NADH–GOGAT activity and total N content decreased, while overexpression of alfalfa NADH–GOGAT in transgenic tobacco increased the N content [27,28].

The pear ranks among the world’s five largest fruits. *Pyrus betulaefolia* (*P.be*), a wild pear species native to China, exhibits strong compatibility with Asian pears in grafting, making it a popular rootstock choice in China [29]. Annually, nitrogen fertilizer application can reach up to 569.60 Kg/ha [30], a figure that continues to rise due to unscientific management. This escalation not only increases grower costs but also contributes to environmental pollution. Despite extensive research on pear dwarfing [29,31], drought resistance [32], and salt resistance [33], enhancing N utilization rate remains an understudied area. This study identified *GS* and *GOGAT* gene family members in the whole genomes of *P.be*, *Pyrus pyrifolia* (*P.py*), and *Pyrus bretschneideri* (*P.br*), followed by bioinformatics analysis. We also examined expression profiles in various *P.be* tissues, gene responses to plant hormones and abiotic stress, and changes in physiological indicators. These findings establish a foundation for future studies of *GS* and *GOGAT* family genes in pears and pave the way for molecular breeding aimed at developing NUE pear rootstocks.

## 2. Results

### 2.1. Identification and Physicochemical Properties of GS and GOGAT Genes in P.be, P.br, and P.py

**Figure 1:**
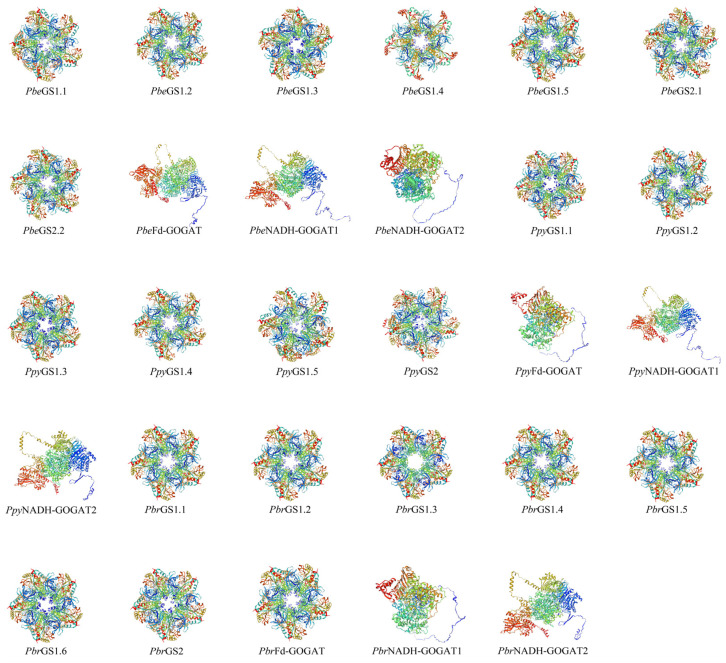
Predicted three–dimensional structures of *GSs* and *GOGATs* proteins in *P.be*, *P.py*, and *P.br*.

Based on conserved protein domains, 29 members of *GS* and *GOGAT* were identified (Table 1). The 21 *GS* members were divided into 7 *PbeGSs* (5 *GS1* and 2 *GS2*), 6 *PpyGSs* (5 *GS1* and 1 *GS2*), and 8 *PbrGSs* (6 *GS1* and 2 *GS2*). The eight *GOGAT* members were divided into three *PbeGOGATs* (one *Fd*–*GOGAT* and two *NADH*–*GOGAT*), three *PpyGOGATs* (one *Fd*–*GOGAT* and two *NADH*–*GOGAT*), and two *PbrGOGATs* (*Fd*–*GOGAT* and *NADH*–*GOGAT*). Moreover, the lengths of *GSs* ranged from 256 to 432 amino acids, and *GOGATs* from 1570 to 2205 aa. The molecular weights ranged from 27.31 kDa (*PpyGS1.1*) to 47.57 kDa (*PbrGS2.1*) for *GSs*, and 171.34 kDa (*PpyFd*–*GOGAT*) to 242.07 kDa (*PbeNADH*–*GOGAT1*) for *GOGATs*. The predicted subcellular localizations included the cytoplasm for *PbeGS1.4*, *PbeGS1.5*, *PpyGS1.4*, *PbrGS1.1*, *PbrGS1.4*, and *PbrGS1.5*; both chloroplast and mitochondrion for *PbeGS2.1*, *PbeGS2.2*, *PpyGS2*, *PbrGS1.3*, *PbrGS2.1*, and *PbrGS2.2*; and chloroplast for *PpyGS1.1* and all *GOGAT* members. The theoretical pI, aliphatic index, instability index, alpha helix, extended strand, and random coil were similar across *GSs* and *GOGATs* in *P.be*, *P.py*, and *P.br*. The secondary protein structures primarily consisted of random coils and alpha helices, with no beta fold structures present. Furthermore, the tertiary structure is shown in Figure 1.

### 2.2. Phylogenetic Relationships of GS and GOGAT Members

**Figure 2:**
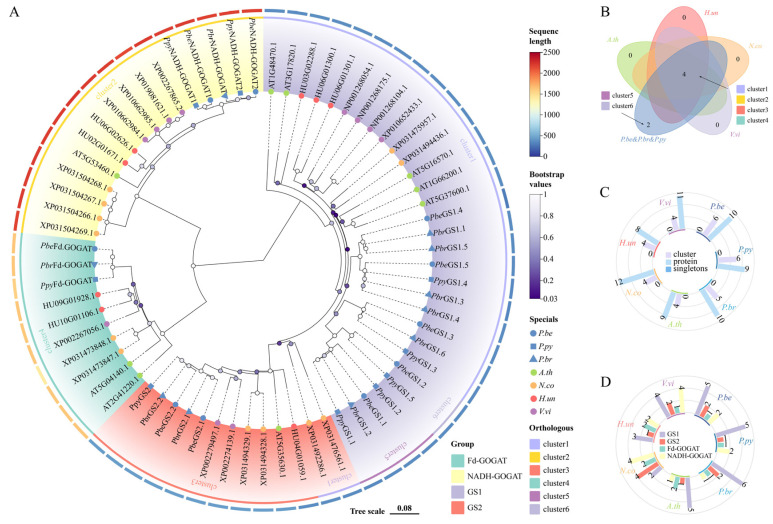
Phylogenetic analysis of pear. (**A**) Phylogenetic analysis of GS and GOGAT in *P.be*, *P.py*, *P.br*, *Arabidopsis thaliana* (*A.th*), *Nymphaea tetragona* (*N.co*), *Hylocereus undatus* (*H.un*), and *Vitis vinifera* (*V.vi*). (**B**) Venn diagram showing the amounts of cluster difference between *P.be*, *P.py*, *P.br*, and the other four species. (**C**) The amounts of cluster, protein, and singletons of GS and GOGAT members of seven species. (**D**) The amounts of GS and GOGAT members of seven species. The percentage of replicate trees in which the associated taxa clustered together in the bootstrap test (1000 replicates) is shown next to the branches.

To elucidate the phylogenetic relationships among *GS* and *GOGAT* members, a phylogenetic tree was constructed using 69 conserved domain sequences from the *GSs* and *GOGATs* proteins of *P.be* (10), *P.br* (10), *P.py* (9), *Arabidopsis thaliana* (*A.th*; 9), *Nymphaea tetragona* (*N.co*; 12), *Hylocereus undatus* (*H.un*; 8), and *Vitis vinifera* (*V.vi*; 11) (Figure 2A). The tree revealed that *GSs* and *GOGATs* from all selected species formed four groups: *GS1* and *GS2* for *GSs*, and *Fd*–*GOGAT* and *NADH*–*GOGAT* for *GOGATs*. Notably, *V.vi GS2s* clustered with those of *P.be*, *P.py*, and *P.br*, reflecting the close relationship between pear and *V.vi*. Notably, the *GSs* and *GOGATs* were further divided into six clusters, each containing at least four members (Figure 2B). Moreover, there are six, six, and five clusters of *P.be*, *P.py*, *P.br*, with *N.co* having the largest members by 12 (Figure 2C). Both *GSs* and *GOGATs* were present in all species (Figure 2D), but the number of gene family members varied. For example, *P.be* had five *GS1*, two *GS2*, one *Fd*–*GOGAT*, and two *NADH*–*GOGAT* members, while *V.vi* had four *GS1*, two *GS2*, one *Fd*–*GOGAT*, and four *NADH*–*GOGAT* members.

### 2.3. Chromosomal Analysis and Collinearity Analysis of Pear GOGAT Members

**Figure 3:**
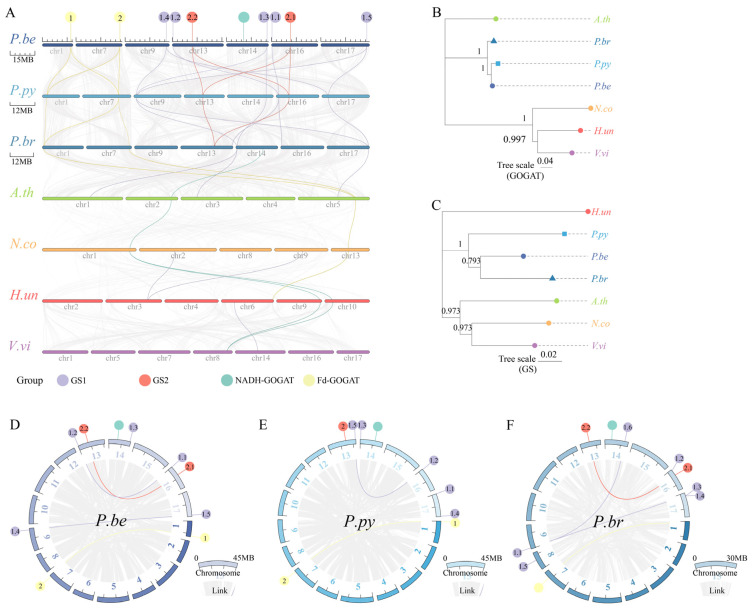
Synteny analysis of GSs and GOGATs. (**A**) Synteny analysis of *GSs* and *GOGATs* in *P.be*, *P.py*, *P.br*, *A.th*, *N.co*, *H.un*, and *V.vi* genomes (purple lines, red lines, green lines, and yellow lines highlight syntenic *GS1s*, *GS2s*, *Fd–GOGATs*, and *NADH–GOGATs* gene pairs, respectively). (**B**) *GOGATs* evolutionary tree of seven species. (**C**) *GSs* evolutionary tree of seven species. (**D**) Synteny analysis of *GSs* and *GOGATs* in *P.be*. (**E**) Synteny analysis of *GSs* and *GOGATs* in *P.py*. (**F**) Synteny analysis of *GSs* and *GOGATs* in *P.br*. Purple and red lines indicate duplicated *GSs* and *GOGATs* gene pairs, and gray lines indicate collinear blocks in the whole *P.be*, *P.py*, and *P.br* genome, respectively.

To elucidate the evolutionary relationship, collinearity analysis was conducted on *P.be*, *P.br*, *P.py*, *A.th*, *N.co*, *H.un*, and *V.vi* (Figure 3A). This analysis identified 27 orthologous gene pairs, including 16 pairs between *P.be* and *P.py* and 11 pairs between *P.py* and *P.br*. However, only six orthologous gene pairs were found between *P.br* and *A.th*. The evolutionary relationships of *GS* and *GOGAT* genes in the seven species revealed a closer relationship among *P.be*, *P.py*, and *P.br* (Figure 3B,C). In these three species, *GS* genes were located on chromosomes 13, 16, and 17. Additionally, *GSs* genes were on chromosomes 9 and 14 in *P.be* (Figure 3D), chromosome 14 in *P.py* (Figure 3E), and chromosomes 8 and 9 in *P.br* (Figure 3F). The *NADH*–*GOGATs* were distributed on chromosomes 1 and 7, while *Fd*–*GOGATs* were on chromosome 14 in *P.be*, *P.py*, and *P.br*. Furthermore, there were four, two, and four orthologous gene pairs resulting from segmental duplication in *P.be*, *P.py*, and *P.br*, respectively.

### 2.4. Conserved Motif and Gene Structure Analyses of GSs and GOGATs

**Figure 4:**
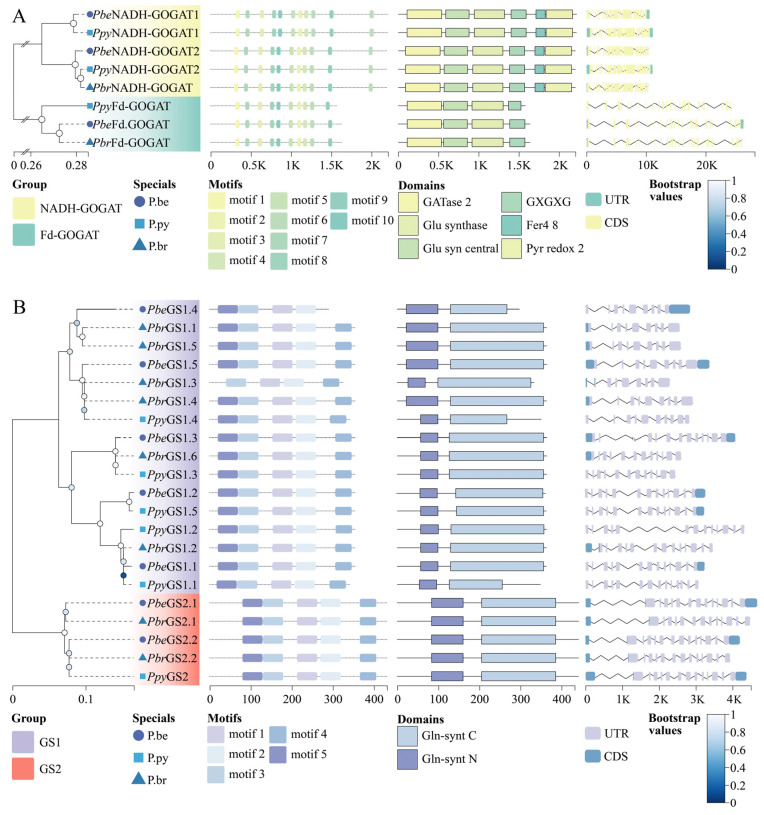
Conserved motif analysis and gene structure analysis of *P.be*, *P.py*, and *P.br*. (**A**) Conserved motif analysis and gene structure analysis of *GOGAT members*. (**B**) Conserved motif analysis and gene structure analysis of *GS* members.

Conserved domains and motifs of *GOGATs* (Figure 4A) and *GSs* (Figure 4B) in *P.be*, *P.py*, and *P.br* were analyzed. All *GS* members exhibited two conserved domains and motifs 1, 2, 3, 4, and 5, except for *PbeGS1.4*, which contained motifs 1, 2, 3, and 5. *Fd*–*GOGATs* and *NADH*–*GOGATs* exhibited six and four conserved domains, respectively, with conserved motifs 1–10 present in all members; *NADH*–*GOGATs* had two motif 6 regions. The number of introns ranged from 8 to 13 in *GS1s*, with *PbeGS1.4*, *PbrGS1.1*, *PbrGS1.5*, *PbeGS1.5*, *PbrGS1.3*, and *PbrGS1.4* having 8 introns, and *PbrGS1.2* having 13 introns. All *GS2s* and *Fd*–*GOGATs* members had 13 and 32 introns, respectively. For *NADH*–*GOGATs*, all members except *PbrNADH*–*GOGAT* had 22 introns, with the exception having 21 introns.

### 2.5. Cis–Acting Elements Were Present in the Promotor Regions of GSs and GOGATs

**Figure 5:**
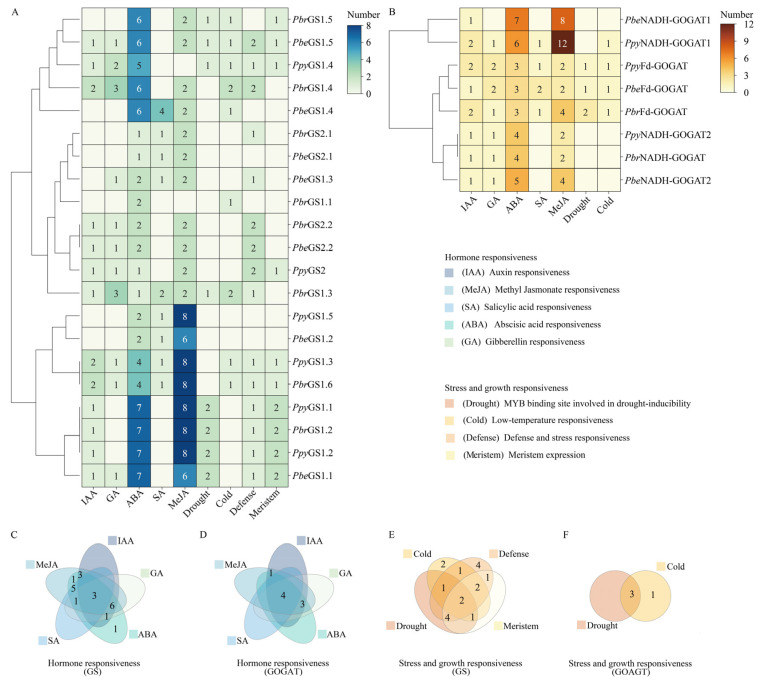
Promoter cis–regulatory element analysis of *GSs* and *GOGATs* in *P.be*, *P.py*, *P.br*. (**A**) The cis–acting elements in the promoter region of *GSs*. (**B**) The cis–acting elements in the promoter region of *GOGATs* (the data in blocks represent the number of cis–elements). (**C**) The amounts of cis–acting elements respond to the hormone responsiveness of *GSs*. (**D**) The amounts of cis–acting elements respond to the hormone responsiveness of *GOGATs*. (**E**) The amounts of cis–acting elements respond to the stress and growth responsiveness of *GSs*. (**F**) The amounts of cis–acting elements respond to the stress and growth responsiveness of *GOGATs*.

From the analysis of cis–elements in the promoter regions of *GSs* (Figure 5A) and *GOGATs* (Figure 5B), we identified elements responsive to hormones such as auxin (IAA), gibberellin (GA), salicylic acid (SA), abscisic acid (ABA), and methyl jasmonate (MeJA), as well as those associated with stress and growth, including MYB binding sites for drought, low–temperature (cold), defense, and stress and meristem–specific expression. Among the *GSs*, *PbrGS1.4*, *PbrGS1.6*, and *PpyGS1.3* had two IAA responsive elements, while *PbrGS1.3* and *PbrGS1.4* had three GA responsive elements. In the *GOGATs* family, all members demonstrated IAA responsiveness (1–2), ABA responsiveness (3–7), and MeJA responsiveness (2–12). Specifically, only *Fd*–*GOGAT* of *P.be*, *P.py*, and *P.br* exhibited drought responsiveness. Additionally, three *GS* members (Figure 5C) and four *GOGAT* members (Figure 5D) possess cis–elements responsive to IAA, GA, SA, ABA, and MeJA. Furthermore, two *GS* members (Figure 5E) exhibit cis–elements for drought, cold, defense, and meristem, while three *GOGAT* members (Figure 5F) have cold– and drought–responsive cis–elements, respectively.

### 2.6. Expression Patterns of PbeGSs and PbeGOGATs Genes in Different Tissues of P.be

**Figure 6:**
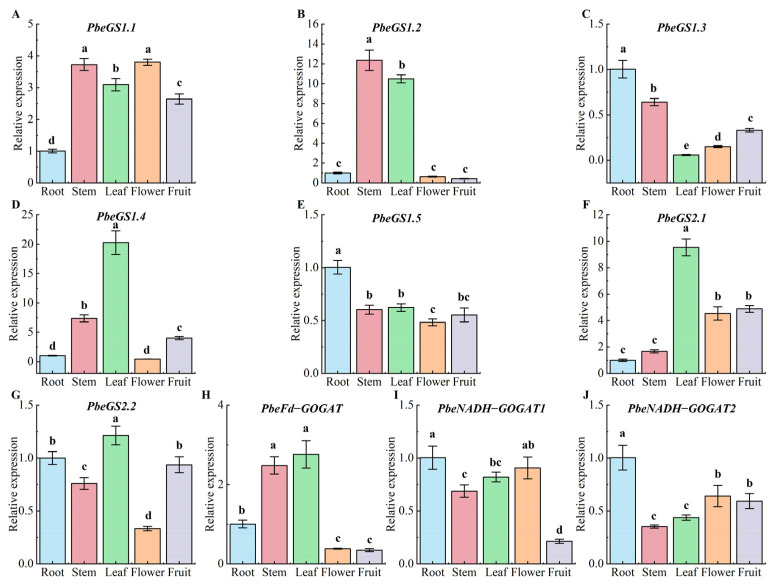
Relative expression analysis of *PbeGSs* and *PbeGOGATs* in different tissues of *P.be*. (**A**) The expression of *PbeGS1.1*. (**B**) The expression of *PbeGS1.2*. (**C**) The expression of *PbeGS1.3*. (**D**) The expression of *PbeGS1.4*. (**E**) The expression of *PbeGS1.5*. (**F**) The expression of *PbeGS2.1*. (**G**) The expression of *PbeGS2.2*. (**H**) The expression of *PbeFd*–*GOGAT*. (**I**) The expression of *PbeNADH*–*GOGAT1*. (**J**) The expression of *PbeNADH*–*GOGAT2*. Each box represents the mean ± SE of three biological replicates (each having three technical replicates). Different letters indicate significant differences, and the same letters represent no significant difference at *p* < 0.05 analyzed by Duncan’s multiple range test.

To investigate *GS* and *GOGAT* expression in pears, we analyzed *PbeGS* and *PbeGOGAT* genes across roots, shoots, leaves, flowers, and young fruits, varying expression levels (Figure 6). Specifically, *PbeGS1.3* (Figure 6C), *PbeGS1.5* (Figure 6E), *PbeNADH*–*GOGAT1* (Figure 6I), and *PbeNADH*–*GOGAT2* (Figure 6J) were highly expressed in roots, while *PbeGS1.4* (Figure 6D), *PbeGS2.1* (Figure 6F), and *PbeGS2.2* (Figure 6G) were highly expressed in the leaves. *PbeGS1.1* (Figure 6A) was highly expressed in flowers. Notably, *PbeGS1.2* (Figure 6B) and *PbeFd*–*GOGAT* (Figure 6H) were highly expressed in both stems and leaves.

### 2.7. Expression Profile Analysis of PbeGSs and PbeGOGATs under Exogenous Hormone, Different NO_3_^−^ Concentrations, and Salt Stress

**Figure 7:**
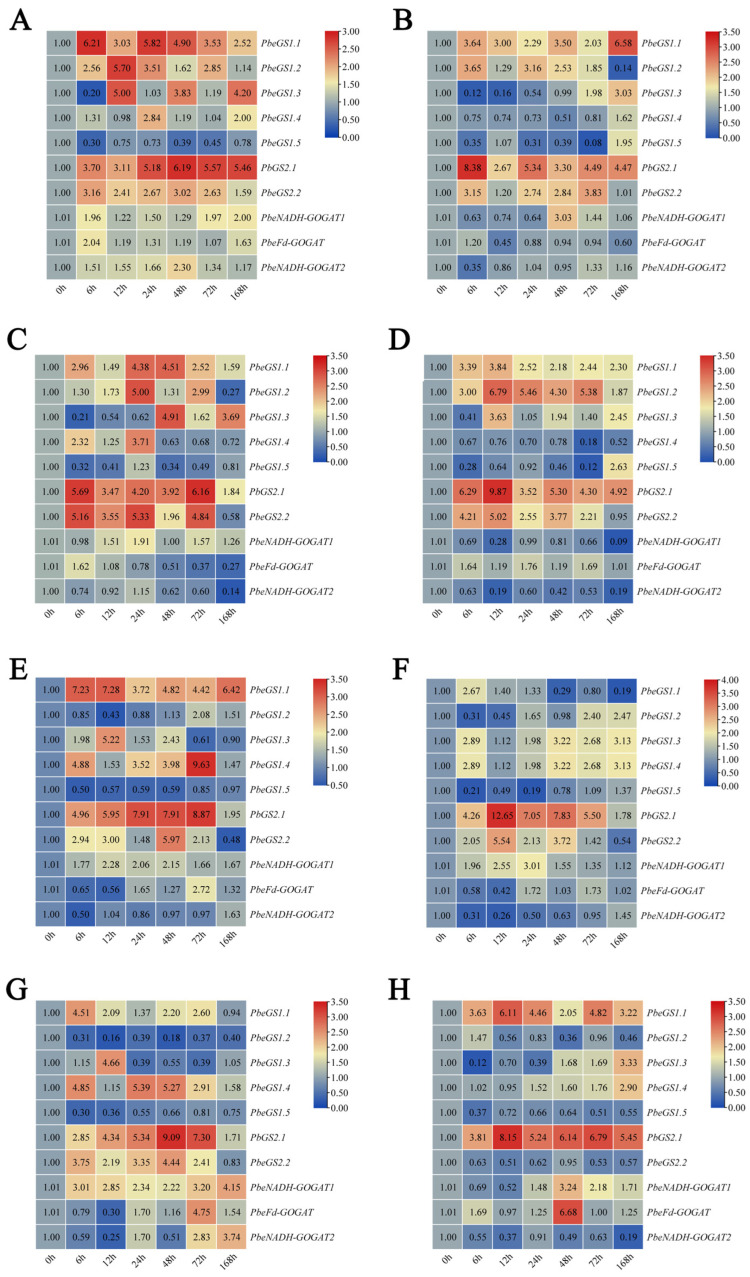
Relative expression analysis of *PbeGSs* and *PbeGOGATs* of *P.be* under exogenous hormone, different NO_3_^−^ concentrations, and salt stress. (**A**) GA_3_ treatment. (**B**) IAA treatment. (**C**) SA treatment. (**D**) ABA treatment. (**E**) 0.5 mM NO_3_^−^ treatment. (**F**) 16 mM NO_3_^−^ treatment. (**G**) 64 mM NO_3_^−^ treatment. (**H**) NaCl treatment. Each box represents the mean ± SE of three biological replicates (each having three technical replicates).

To clarify the role of *GS* and *GOGAT* members under exogenous hormones and abiotic stress, we examined 10 members of the *PbeGS* and *PbeGOGAT* gene families. Under GA_3_ treatment (Figure 7A), all genes except *PbeGS1.3* and *PbeGS1.5* were significantly upregulated. *PbeGS1.1*, *PbeGS2.2*, *PbeFd*–*GOGAT*, and *PbeNADH*–*GOGAT1* showed the highest expression levels at 6 h, increasing by 6-, 3-, 2-, and 2-fold compared to 0 h, respectively. Conversely, *PbeGS1.3* and *PbeGS1.5* decreased to their lowest, 0.2– and 0.3–fold of their initial levels at 6 h. Under IAA treatment (Figure 7B), most genes, including *PbeGS1.3*, *PbeGS1.4*, *PbeGS1.5*, *PbeNADH*–*GOGAT1*, and *PbeNADH*–*GOGAT2*, first decreased and then increased. *PbeGS1.3*, *PbeGS1.4*, and *PbeGS1.5* reached their highest expression levels at 168 h. When treated with SA (Figure 7C), *PbeGS1.2*, *PbeGS1.4*, *PbeGS2.2*, and *PbeFd*–*GOGAT* were initially upregulated and then downregulated, with all except *PbeFd*–*GOGAT* peaking at 24 h. *PbeGS1.3*, *PbeGS1.5*, and *PbeNADH*–*GOGAT2* were down–regulated at 6 h, showing time–dependent variations in expression. In ABA treatment (Figure 7D), *PbeGS1.1*, *PbeGS1.2*, *PbeGS2.1*, *PbeGS2.2*, and *PbeFd*–*GOGAT* were up–regulated, reaching peak expression at 12 h with increases of 4-, 7-, 10-, and 5-fold, respectively, except for *PbeFd*–*GOGAT*. In contrast, *PbeGS1.4*, *PbeNADH*–*GOGAT1*, and *PbeNADH*–*GOGAT2* were down–regulated.

Under 16 mM NO_3_^−^ treatment (Figure 7F), *PbeGS1.1*, *PbeGS1.4*, *PbeGS2.1*, and *PbeNADH*–*GOGAT1* expression increased, while *PbeGS1.2*, *PbeGSFd*–*GOGAT*, and *PbeNADH*–*GOGAT2* were initially downregulated and subsequently upregulated. Under abiotic stress, *PbeGS1.4*, *PbeGS2.1*, and *PbeNADH*–*GOGAT1* expression levels were up–regulated under 0.5 mM NO_3_^−^ (Figure 7E), 64 mM NO_3_^−^ (Figure 7G), and NaCl treatments (Figure 7H), whereas *PbeFd*–*GOGAT* expression initially decreased and then increased. *PbeNADH*–*GOGAT2* expression also decreased initially before increasing under 0.5 mM NO_3_^−^ and 64 mM NO_3_^−^ treatments. However, there were differences between treatments. For example, *PbeGS1.2* expression initially decreased and then increased under 0.5 mM NO_3_^−^ treatment, but increased first and then decreased under NaCl treatment. *PbeGS2.2* expression was upregulated under 64 mM NO_3_^−^, but decreased under NaCl treatment, showing expression levels 0.3- and 2-fold compared to 0 h, respectively.

### 2.8. Effects of Exogenous Hormones, Different NO_3_^−^ Concentrations, and Salt Stress on Chlorophyll Content, Enzyme Activity, and N Content of P.be

**Figure 8:**
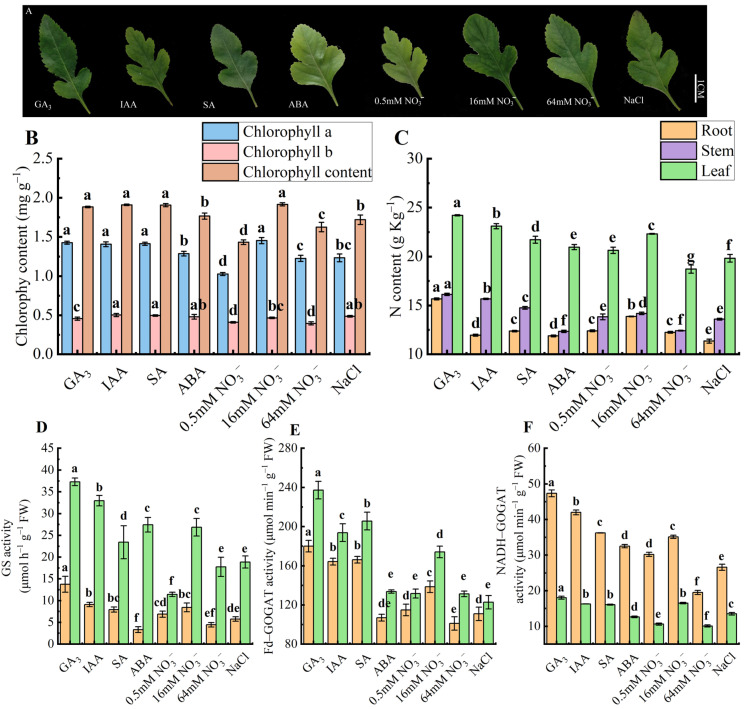
Effects of exogenous hormones, different NO_3_^−^ concentrations, and salt stress on chlorophyll, enzyme activity, and N content of *P.be*. (**A**) Leaf phenotypes. (**B**) The content of chlorophyll. (**C**) The content of N. (**D**) The activity of GS. (**E**) The activity of Fd–GOGAT. (**F**) The activity of NADH–GOGAT. Each box represents the mean ± SE of three biological replicates (each having three technical replicates). Different letters indicate significant differences, and the same letters represent no significant difference at *p* < 0.05 (n = 3) analyzed by Duncan’s multiple range test.

The chlorophyll content of *P.be* leaves varied under different treatments (Figure 8A). The chlorophyll a and total chlorophyll contents significantly (*p* < 0.05) decreased with ABA, 0.5 mM NO_3_^−^, and 64 mM NO_3_^−^ treatments, while chlorophyll b significantly increased with IAA and SA treatments compared to the 16 mM NO_3_^−^ treatment (Figure 8B). The N content (Figure 8C) in leaves, roots, and stems significantly increased under GA_3_ and IAA treatments, with increases ranging from 3.51% to 26.67%, whereas it significantly decreased with ABA, 0.5 mM NO_3_^−^, and NaCl treatments. Notably, N content in leaves and stems significantly increased under GA_3_ and IAA treatments. The enzymatic activity showed similar performance under different treatments; GS (Figure 8D) and NADH–GOGAT (Figure 8F) significantly increased under GA_3_ treatment, but decreased by 14.01–57.44% under 0.5 mM NO_3_^−^ and 64 mM NO_3_^−^ compared to 16mM NO_3_^−^ treatment. Additionally, the Fd–GOGAT (Figure 8E) significantly increased in leaves and roots under GA_3_, IAA, and SA treatments, with increases from 11.34% to 36.28%.

### 2.9. Correlation Analysis

**Figure 9:**
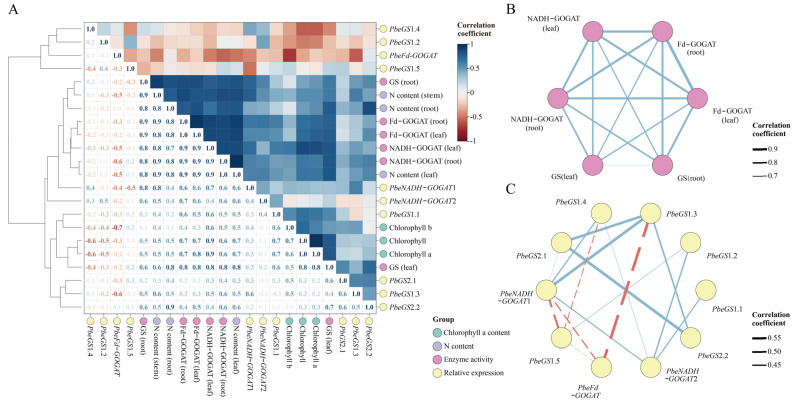
Correlation analysis of gene expression and plant physiology under exogenous hormones and abiotic stresses at 168 h. (**A**) Correlation matrix heat map based on 22 characters of gene expression and physiological indexes. (**B**) Correlation matrix based on the activity of GS and GOGAT in leaves and roots. (**C**) Correlation matrix based on *PbeGS* and *PbeGOGAT* gene expression level in leaves. The blue solid line represents the positive correlation and the red dashed line represents the negative correlation.

According to the correlation matrix, chlorophyll a and total chlorophyll content were significantly positively correlated (*p* > 0.5) with the leaf N content (Figure 9A). The Fd–GOGAT, NADH–GOGAT, and GS activities in both leaves and roots were significantly correlated with N content in roots, stems, and leaves. Additionally, the expression levels of *PbeGS1.1* and *PbeGS2.2* were significantly positively correlated with N content, Fd–GOGAT, and GS in leaves. Both *PbeNADH*–*GOGAT1* and *PbeNADH*–*GOGAT2* expression were significantly positively correlated with N content and Fd–GOGAT in the leaves. However, *PbeFd*–*GOGAT* expression was significantly negatively correlated with N content in the leaves and NADH–GOGAT activity in the roots. Meanwhile, the enzymatic activity (Figure 9B) showed significant positive correlations among these indices. Interestingly, the expression level of *PbeGS2.1* was significantly positively correlated with *PbeGS1.3* and *PbeGS2.2* (Figure 9C).

## 3. Discussion

From this study, we found there was a significant positive correlation between the GS and GOGAT activities and N content in *P.be* seedlings, which has also been reported in other plants [34]. Therefore, identifying GS and GOGAT family members can accelerate the cultivation of NUE pear rootstocks. However, the study of *GSs* and *GOGATs* functions in pear is limited, necessitating their identification in this species. Tandem duplication, segmental duplication, and whole–genome duplication are key drivers of plant evolution [29]. This study identified two *GS2* members in *P.be* and *P*.*br*, whereas most plants have only one *GS2* member [35]. This might be due to gene duplication differences. The pear *GOGATs*, including *Fd*–*GOGAT* and *NADH*–GOGAT, are consistent with those in most higher plants [36]. Additionally, there were 3, 2, and 4 pairwise genes in *P.be*, *P.py*, and *P.br*, respectively, such as *PbeGS1.4* and *PbeGS1.5*, *PpyGS1.2* and *PpyGS1.5*, and *PbrGS2.1* and *PpyGS2.2.* Additionally, these closely related genes on the evolutionary tree suggest gene segmental duplication during evolution, which likely drives gene–family expansion [32,37,38]. Meanwhile, most *GS* and *GOGAT* members of *P.be*, *P.py*, and *P.br* cluster more closely together than those of other species within the same clusters. This indicates strong conservatism within the *GS* and *GOGAT* gene families among pear species.

Intron numbers play a crucial role in gene evolution [39]. Our analysis of *GSs* and *GOGATs* gene structures revealed that nearly all *GS1* genes of *P.be*, *P.py*, and *P.br* contained different numbers of introns, indicating functional diversity among these *GS1* genes. In contrast, the *GS2s* (13), *NADH*–*GOGATs* (21), and *Fd*–*GOGATs* (32) had the same number of introns, suggesting similar functionality. Cis-regulatory elements likely allow plants to influence growth and development in various ways [39]. Analysis of cis–regulatory elements identified a series of hormone– and abiotic stress–responsive elements in the promoter regions of GS and GOGAT genes, including GA, SA, IAA, ABA, MeJA, and drought stress–core elements. This indicates that *GSs* and *GOGATs* may function in response to hormones and abiotic stresses.

In many plants, members of the *GS* and *GOGAT* gene families exhibit spatial and functional specificity [40]. Our study revealed that *PbeGS2s* and *PbeFd*–*GOGAT* are highly expressed in leaves, while *PbeGS1.3*, *PbeGS1.5*, and *PbeNADH*–*GOGATs* show high expression in roots, consistent with findings in *Populus* [22] and Cucurbitaceae [35]. *GS2* primarily assimilates ammonia produced by nitrate reduction and photorespiration in leaves, whereas *GS1* assimilates NH_4_^+^ from other metabolic processes [9]. *Fd*–*GOGAT* mainly assimilates NH_4_^+^ from photorespiration in photosynthetic tissues, while *NADH*–*GOGAT* handles NH_4_^+^ assimilation in non–photosynthetic tissues [41]. Therefore, gene expression varies across tissues. Notably, *PbeGS1.2*, which is also highly expressed in stems, suggests a crucial role in intercellular nitrogen transport. These results indicated that *PbeGS* and *PbeGOGAT* genes play diverse roles during pear growth and tissue development.

The activities of GS and GOGAT are crucial for plant growth and development [22]. The results showed exogenous hormones effectively regulated the activity of GS and GOGAT. In which, the GS activity increased under GA_3_ and IAA treatment. The reason may be that GA_3_ and IAA increased the expression level of *PbeGS1.1*, *PbeGS1.2*, *PbeGS2s*, and *PbeGOGATs*. The same results were also found in maize [17], *Arabidopsis* [42], soybean [43], and other plants [22]. Previous studies have reported that exogenous GA_3_ both promotes the expression level of *GS* and *GOGAT* in ‘duli’ [44], and GA_3_ increases the expression level of *OsGS1.2* and increases the activity of GS in rice [45]. This may be caused by the presence of hormone–responsive elements in the gene. Meanwhile, the over–expression of *GS1.2* increases the content of IAA in tobacco [46], which suggested that there may be an interaction between the expression of *GS* and the synthesis of IAA. Appropriate N application boosts GS and GOGAT activities and their expression levels, whereas N stress has the opposite effect. For example, high N stress reduces GS activity in rice [45]. In *Malus*, N stress affects the expression levels of *GS* and *GOGAT* [47]. Under low N stress conditions, GS and GOGAT activities and expression levels are induced in rice [48]. From our study, we found both GS and GOGAT activities decreased under 0.5 mM and 64 mM NO_3_^−^ treatments. The expression levels of *PbeGS1.1* and *PbeGS2.2* initially increased and then decreased under 0.5 mM NO_3_^−^ treatment, indicating these genes may be crucial in NO_3_^−^ stress response. This is consistent with previous studies [49,50]. The main reason is that, under low N stress, plants may reuse stored N by degrading N compounds to survive [51]. *PbeGS1.1* may function in the utilization of this recycled N. Similarly, we also found GS and GOGAT activities decreased under NaCl treatment, with a notable reduction in Fd–GOGAT. This may be the main reason for the decrease in GOGAT enzyme activity, due to the fact that Fd–GOGAT activity accounts for 95% of GOGAT activity in leaves [52]. The study also revealed a decrease in chlorophyll content, which may explain the reduced Fd–GOGAT activity and expression in leaves under NaCl stress [53]. Additionally, Fd–GOGAT activity surpassed NADH–GOGAT activity, and GS activity was higher in leaves than in roots, consistent with previous findings [54].

The correlation analysis revealed a significant positive correlation between *PbeGS2.1* and *PbeGS2.2* under exogenous hormones and abiotic stresses, indicating functional redundancy among these genes. Conversely, *PbeGS1.3* exhibited a negative correlation with *PbeFd*–*GOGAT*, suggesting distinct roles under similar conditions. Therefore, the *GS* and *GOGAT* families possess crucial potential functions for growth and abiotic stress response. However, the specific functions of *PbeGSs* and *PbeGOGATs* require further in–depth investigation.

## 4. Materials and Methods

### 4.1. Plant Materials and Treatment

Five tissues were collected from roots, stems, leaves, flowers, and young fruits at various times during the growing season from three 5-year-old *Pyrus betulifolia* (*P.be*) trees maintained in the Resource Orchard of Hebei Agricultural University, Hebei Baoding, China. The stem segments (1–2 cm) from the same trees were used as explants to generate tissue–cultured seedlings. Root culture was established, and seedlings with roots were transplanted into 7 × 7 × 7 cm bowls, one seedling per bowl, using a vermiculite and perlite medium in a 1:1 ratio. The seedlings were irrigated with 1/2 Hoagland solution every three days to ensure normal growth, following the Hoagland nutrient solution protocol of Chen et al. (2018) [55]. After one month, 400 healthy and similarly–sized seedlings were divided into 8 groups of 50. Treatments included 0.1 mM GA_3_ [44], 0.1 mM IAA, 0.2 mM SA [56], 0.3 mM ABA [57], and 200 mM NaCl [58]. Previous reports have indicated that 16 mM NO_3_^−^ (Ca(NO_3_)_2_) is the optimal nitrogen concentration; 0.5 mM NO_3_^−^ (Ca(NO_3_)_2_) was deficit stress, and 64 mM NO_3_^−^ (Ca(NO_3_)_2_) was supraoptimal stress [55]. Each treatment was applied for 168 h, with irrigation every 72 h. *P.be* leaves were sampled at 0 h, 6 h, 12 h, 24 h, 48 h, 72 h, and 168 h. The samples were immediately frozen in liquid nitrogen and stored at −80 °C for analysis.

### 4.2. Identification and Physicochemical Analysis of the GS and GOGAT Family in P.be, P.br, and P.py

The *A.th* database (https://www.arabidopsis.org/, accessed on 10 August 2023) was utilized to search for *GS* and *GOGAT* gene family members. The *P.be*, *P.py*, and *P.br* genome sequences were retrieved from the Genome Database for Rosaceae of *Pyrus betulifolia*, ‘Cuiguan’, and ‘Dangshansuli’, respectively (https://www.rosaceae.org/, accessed on 10 August 2023). These pear genome sequences were used to construct a local BLAST database, with *A.th GS* and *GOGAT* protein sequences serving as target sequences for a local BLAST search (E ≤ 1 × 10^−5^). HMMER3.0 software was employed to eliminate redundant sequences. Further screening was conducted using SMART (http://smart.emblheidelberg.de/, accessed on 20 May 2023) and NCBI–CDD (https://www.ncbi.nlm.nih.gov/Structure/cdd/wrpsb, accessed on 20 May 2023). *GS* candidate genes were then verified to confirm the domains Gln–synt–C (PF00120) and Gln–synt–N (PF03951), while *GOGAT* candidates were verified for the CATase–2 (PF00310), Glu–synthase (PF01645), Glu–sy–central (PF04898), and GXGXG (PF01493) domains.

The physical and chemical properties of *P.be*, *P.br*, and *P.py* proteins (length, molecular weight, isoelectric point, fat coefficient, unstable factor) were analyzed using the Expasy proteomics server (https://web.expasy.org/protparam/, accessed on 20 May 2023). Secondary and tertiary structures of these proteins were predicted using the Secondary Structure Prediction tool (https://npsa-prabi.ibcp.fr/cgi-bin/npsa_automat.pl?page=npsa%20_sopma.html, accessed on 20 May 2023) and SWISS–MODEL (https://swissmodel.expasy.org/interactive, accessed on 20 May 2023), respectively. The subcellular localization of GS and GOGAT proteins was determined via an online resource website (http://www.csbio.sjtu.edu.cn/bioinf/Cell-PLoc-2/, accessed on 25 May 2023).

### 4.3. Evolutionary Analysis of the GS and GOGAT Family in P.be, P.br, and P.py

The amino acid sequences of *P.be*, *P.br*, and *P.py* proteins were compared with those of *A.th*, *N.co*, *H.un*, and *V.vi* using Clustal (https://www.ebi.ac.uk/Tools/msa/clustalo/, accessed on 29 May 2023). A phylogenetic tree was then constructed using the neighbor–joining method in MEGA7.0 software (version 7.0, Mega Limited, Auckland, New Zealand).

### 4.4. Analysis of Gene Structure and Conserved Motif

GSDS v2.0 (http://gsds.cbi.pku.edu.cn/, accessed on 26 May 2023) was used to predict the inline exon structure of the *GS* and *GOGAT* gene families in *P.be*, *P.br*, and *P.py*. MEME (http://meme-suite.org/tools/meme, accessed on 26 May 2023) analyzed the motif composition of GS and GOGAT proteins (motif size: 6–50, number: 10, default settings for other parameters), and the results were visualized using the “Gene Structure View (Advanced)” tool in TBtools software (version 2.119, College of Horticulture, South China Agricultural University, China).

### 4.5. Analysis of Synteny and Gene Duplication

To explore the collinear relationship among *P.be*, *P.br*, and *P.py*, genomic data and genome annotation files for *Pyrus*, *A.th*, *N.co*, *H.un*, and *V.vi* were downloaded from the GDR, TAIR, and NCBI databases and visualized with Tbtools.

### 4.6. Prediction and Analysis of Cis-Elements in the Promoter Regions of GSs and GOGATs of P.be

Tbtools was used to extract the upstream 2000 bp sequence of each *GS* and *GOGAT* member. The sequences were submitted to the online website Plant CARE (http://bioinformatics.psb.ugent.be/webtools/plantcare/html/, accessed on 29 May 2023) to predict promoter regions of cis−elements, and the results were visualized using the TBtools Simple BioSequence Viewer feature.

### 4.7. Total RNA Extraction and First–Strand cDNA Synthesis and qRT–PCR Assay

Total RNA extraction was performed using the method described by Song et al. [29]. The first–strand cDNA was synthesized from 2 µg of total RNA using the First–Strand cDNA Synthesis Kit (Yeasen, Shanghai, China). Transcript levels were identified using qRT–PCR (Applied Biosystems, San Francisco, CA, USA). Each 20.0 μL reaction mixture contained 10.0 μL SYBR Supermix (Yeasen, Shanghai, China), 2.0 μL cDNA template, 0.4 μL forward and reverse primers, and 7.2 μL RNA–free H_2_O. Each treatment was replicated three times. qRT–PCR primers were designed with Premier 5.0 software (Premier Biosoft International, Silicon Valley, CA, USA). *PbeActin* was used as the internal reference standard (Supplementary Table S1). The 2^−△△CT^ method was used to calculate the relative expression levels of the target genes.

### 4.8. The Chlorophyll Content, Enzyme Activity, and N Content Measurements

At 168 h, 0.1 g of leaves, ground to powder in liquid nitrogen, were extracted in 10 mL of 80% acetone for over 24 h in the dark. Chlorophyll concentration was determined by measuring light absorption at 663 and 645 nm using a UV–1800 spectrophotometer (UV–1800, Metash, Shanghai, China) [59,60].

The enzyme activities of GS, Fd–GOGAT, and NADH–GOGAT in roots and leaves were measured using their respective commercial kit (Suzhou Geruisi Biotechnology, Suzhou, China) according to the manufacturer’s instructions.

The N content was determined using a continuous flow analyzer (Auto Analyzer 3, SEAL Analytical, Norderstedt, Germany) [61,62]. The *P.be* seedlings were divided into roots, stems, and leaves, washed twice with 1% (*w*/*v*) citric acid, and rinsed three times with deionized water. Samples were fixed at 105 °C for 15 min and oven–dried to a constant weight at 70 °C. For digestion, 0.1 g samples were placed into a tube, soaked in 1 mL ultra–pure water for 1 min, and then mixed with 8 mL of sulfuric acid and perchloric acid (10:1). The mixture was heated to 280 °C until clear and transparent (Multiwave PRO; Anton–Paar GmbH, Graz, Austria). Each treatment included three independent biological replicates.

### 4.9. Statistical Analysis

Gene expression levels, chlorophyll content, enzyme activity, and N content were recorded using Microsoft Excel 2016. These data were then analyzed using SPSS 25 (SPSS Inc., Chicago, IL, USA) through one–way ANOVA and Tukey’s test (*p* < 0.05) to identify significant differences. Figures were generated with Origin 2019 (OriginLab, Northampton, MA, USA). Data are presented as mean ± standard deviation.

## 5. Conclusions

**Figure 10:**
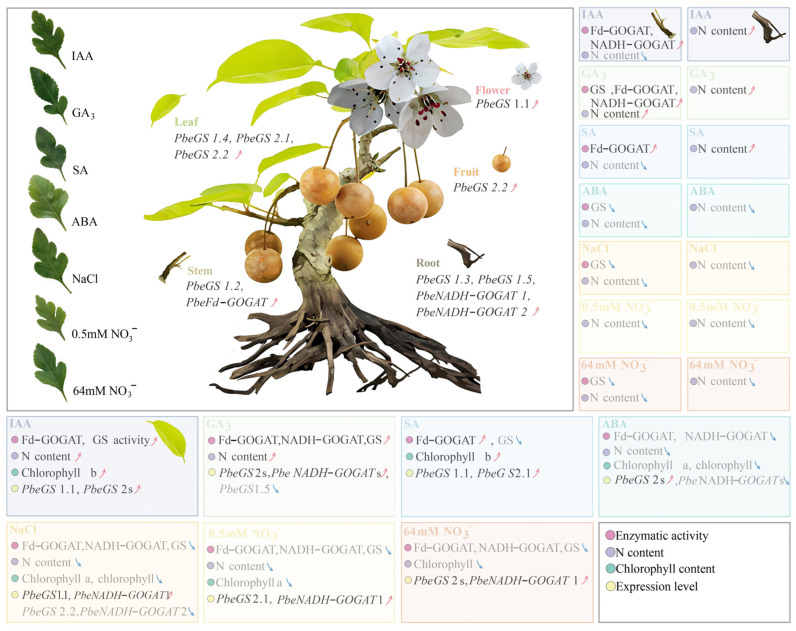
Pattern of *PbeGS* and *PbeGOGAT* genes expression and relative physiology indexes analysis under exogenous hormones and abiotic stresses.

In conclusion, bioinformatic analysis identified seven, six, and seven *GS* members and three, two, and three *GOGAT* members in *P.be*, *P.py*, and *P.br*, respectively. Meanwhile, the *GS* and *GOGAT* families have highly evolutionary conservation in pears, and segmental duplication has driven their expansion and evolution. Additionally, the expression profiles of these genes showed that they were ubiquitously expressed under endogenous hormones and abiotic stress, indicating that these genes may play an important role in the ‘duli’ growth and development and environmental response (Figure 10). Combined with correlation analysis and qRT–PCR, *PbeGS1.1*, *PbeGS2.2*, and *PbeNADH*–*GOGATs* may be considered as potential candidate genes to regulate N metabolism in *P.be* under hormones and abiotic stress conditions.

## Figures and Tables

**Table 1:** Amino acid composition and physiochemical characteristics of GS and GOGAT proteins in *Pyrus betulaefolia* (*P.be*), *Pyrus pyrifolia* (*P.py*), and *Pyrus bretschneideri* (*P.br*).

Name	Protein ID	Length (aa)	CDS Length(bp)	Molecular Weight (kDa)	Theoretical pI	Aliphatic Index	Instability Index	Cell Localization	Alpha Helix (%)	Extended Strand (%)	Beta Turn (%)
*PbeGS1.1*	GWHPAAYT024454	355	1068.00	38.87	6.27	79.21	41.89	Chloroplast. Cytoplasm	26.20	14.65	0.00
*PbeGS1.2*	GWHPAAYT012997	355	1068.00	38.04	5.55	81.97	39.57	Chloroplast. Cytoplasm	28.17	11.83	0.00
*PbeGS1.3*	GWHPAAYT019348	356	1071.00	39.24	5.87	79.49	43.02	Chloroplast. Cytoplasm	27.81	15.17	0.00
*PbeGS1.4*	GWHPAAYT056312	291	876.00	31.69	5.65	83.85	38.32	Cytoplasm	33.33	12.03	0.00
*PbeGS1.5*	GWHPAAYT030844	356	1071.00	39.10	5.94	78.96	38.70	Cytoplasm	27.25	14.61	0.00
*PbeGS2.1*	GWHPAAYT026125	432	1299.00	47.55	6.37	77.71	43.08	Chloroplast. Mitochondrion	23.61	16.44	0.00
*PbeGS2.2*	GWHPAAYT014733	432	1299.00	47.55	6.37	77.94	42.90	Chloroplast. Mitochondrion	25.93	14.81	0.00
*PpyGS1.1*	GWHPBAOS040677	341	1026.00	27.31	7.02	79.59	44.83	Chloroplast	24.34	16.44	0.00
*PpyGS1.2*	GWHPBAOS017647	356	1071.00	38.97	6.02	78.99	41.73	Chloroplast. Cytoplasm	24.72	15.45	0.00
*PpyGS1.3*	GWHPBAOS011637	256	771.00	28.32	6.21	75.12	39.15	Chloroplast. Cytoplasm	28.52	13.28	0.00
*PpyGS1.4*	GWHPBAOS021779	343	1032.00	37.75	5.50	78.83	40.71	Cytoplasm	28.57	13.70	0.00
*PpyGS1.5*	GWHPBAOS011461	355	1068.00	38.82	5.55	82.23	39.09	Chloroplast. Cytoplasm	28.45	12.39	0.00
*PpyGS2*	GWHPBAOS009980	432	1299.00	47.55	6.37	77.94	42.90	Chloroplast. Mitochondrion	25.93	14.81	0.00
*PbrGS1.1*	rna24437	356	1071.00	38.96	5.94	78.96	35.15	Cytoplasm	29.49	12.92	0.00
*PbrGS1.2*	rna5646	355	1068.00	38.87	6.27	80.59	40.93	Chloroplast. Cytoplasm	26.20	15.49	0.00
*PbrGS1.3*	rna24970	326	981.00	35.75	6.12	74.26	37.63	Chloroplast. Mitochondrion	23.62	14.11	0.00
*PbrGS1.4*	rna24969	356	1071.00	39.02	5.78	79.24	37.73	Cytoplasm	27.25	14.33	0.00
*PbrGS1.5*	rna39988	356	1071.00	38.90	5.94	78.68	35.69	Cytoplasm	28.37	13.76	0.00
*PbrGS1.6*	rna14132	356	1071.00	39.25	5.87	79.49	44.63	Chloroplast. Cytoplasm	28.37	16.49	0.00
*PbrGS2.1*	rna41071	432	1299.00	47.57	6.37	78.38	42.74	Chloroplast. Mitochondrion	24.31	16.90	0.00
*PbrGS2.2*	rna6045	432	1299	47.55	6.37	77.94	42.9	Chloroplast. Mitochondrion	25.93	14.81	0.00
*PbeFd*–*GOGAT*	GWHPAAYT017638	1628	4887.00	177.03	6.25	90.15	35.84	Chloroplast	39.62	14.68	0.00
*PbeNADH*–*GOGAT1*	GWHPAAYT002126	2205	6618.00	242.07	6.22	84.12	35.77	Chloroplast	37.05	15.78	0.00
*PbeNADH*–*GOGAT2*	GWHPAAYT050332	2189	6570.00	240.71	6.42	84.33	35.90	Chloroplast	37.32	16.31	0.00
*PpyNADH*–*GOGAT1*	GWHPBAOS000126	2202	6609.00	241.80	6.28	84.33	35.78	Chloroplast	37.19	15.67	0.00
*PpyNADH*–*GOGAT2*	GWHPBAOS035473	2189	6570.00	240.51	6.23	84.19	36.10	Chloroplast	37.14	16.45	0.00
*PpyFd*–*GOGAT*	GWHPBAOS012871	1570	4713.00	171.34	6.05	88.94	36.26	Chloroplast	39.49	13.76	0.00
*PbrNADH*–*GOGAT*	rna33473	2190	6573.00	240.56	6.23	84.42	36.17	Chloroplast	37.20	16.44	0.00
*PbrFd*–*GOGAT*	rna37178	1628	4887.00	177.01	6.34	90.09	35.50	Chloroplast	39.31	14.77	0.00

## Data Availability

Data are contained within the article and Supplementary Materials.

## Supplementary Materials

The following supporting information can be downloaded at: https://www.mdpi.com/article/10.3390/plants13192759/s1, Table S1 Sequences of primers used in qRT–PCR.
