# Supplementary material for: Glutamine Synthetase and Glutamate Synthase Family Perform Diverse Physiological Functions in Exogenous Hormones and Abiotic Stress Responses in Pyrus betulifolia Bunge (P.be)
Source: Plants (Basel). 2024 Oct 1;13(19):2759. doi: 10.3390/plants13192759 (PMC11479100; doi:10.3390/plants13192759)
Supplement: Supplementary file 1 [file plants-13-02759-s001.zip › plants-3238423-supplementary.pdf]

Glutamine Synthetase and Glutamate Synthase Family Perform Diverse Physiological Functions in Exogenous Hormones and Abiotic Stress Responses in *Pyrus betulifolia* Bunge (*P.be*)

Weilong Zhang[1], Shuai Yuan[1], Na Liu[1,2], Haixia Zhang[1,2,*] and Yuxing Zhang[1,2,*]

1 College of Horticulture, Hebei Agricultural University, Baoding, 071001, China
2 Pear technology and innovation center of Hebei Province, Baoding, 071001, China
* Correspondence: zhx2323a@163.com, zhyx@hebau.edu.cn.

Supplementary Data

Table S1 Sequences of primers used in qRT−PCR.

Gene	Forward primer sequence (5'−3')	Reverse primer sequence (5'−3')
PbeGS1.1	GGACCAGTAACAGACCCAGC	CGCATCGCATATCACCAGGA
PbeGS1.2	AGAAACTGAGTCTGCGCCAC	TCAGTTTCACGACCAACCCG
PbeGS1.3	ATGTCGTAGCCGAAGAACCC	TCACGCCCAAAGGACTTCTC
PbeGS1.4	TCGACCTCTCAGACAGCACT	GATCACTCACTGGTCCTGGC
PbeGS1.5	CGCACTACAAGGCCTGTCTC	GAGCTGCCCACAGTTCATCT
PbeGS2.1	AAACCGATAGAGGGTGACTGG	TCACCGTGGCGAAGTGACA
PbeGS2.2	ATTTGGATCGGAGGGTCAGG	GTCCGGTACTTGATCCATCGT
PbeFd−GOGAT	TAAATCGAAGCGGACTCGCA	AGTGGCAATTCCTGGAGACG
PbeNADH−GOGAT1	GCGACGGTAGCAGAGAAGTT	CTTCCTCCTCGTTCAAGTCGT
PbeNADH−GOGAT2	CAGGGGCTAAGGCAGTTGTC	TGTGGCGTTGATGTTGTTGTAT
PbActin	TTGGTATGGGTCAGAGG	CTGTGAGCAGAACTGGGTG
